# Host immunity and HBV S gene mutation in HBsAg-negative HBV-infected patients

**DOI:** 10.3389/fimmu.2023.1211980

**Published:** 2023-08-14

**Authors:** Xin Liu, Shu-xiang Chen, Hui Liu, Jin-li Lou

**Affiliations:** Department of Clinical Laboratory Center, Beijing Youan Hospital, Capital Medical University, Beijing, China

**Keywords:** T lymphocyte, cytokine, HBsAg negative, HBV DNA, Occult hepatitis B virus infection

## Abstract

**Background:**

Clinically, some patients whose HBsAg becomes negative owing to antiviral therapy or spontaneously still show a low level of HBV DNA persistence in serum. T-lymphocyte subsets, cytokine levels and HBV S gene sequences were analyzed in this study.

**Methods:**

A total of 52 HBsAg-negative and HBV DNA-positive patients(HBsAg-/HBV DNA+ patients), 52 persistently HBsAg-positive patients(HBsAg+/HBV DNA+ patients) and 16 healthy people were evaluated. T-lymphocyte subsets of these patients were detected by flow cytometry, serum cytokines and chemokines were detected by the Luminex technique, and the HBV S region was evaluated by Sanger sequencing. T%, T-lymphocyte, CD8+ and CD4+T lymphocyte were lower in the HBsAg-negative group than in the HC group. Compared with the HBsAg-positive group, the HBsAg-negative group had lower levels in T lymphocyte %, CD8+T lymphocyte %, CD8+T lymphocyte and CD4/CD8. These difference were statistically significant (*P*<0.05). Serum IFN-γ, IFN-α and FLT-3L levels were significantly higher in the HBsAg-negative group than in the HBsAg-positive group (*P*<0.05). However, levels of many cytokines related to inflammation (i.e., IL-6, IL-8, IL10, IL-12, IL-17A) were lower in the HBsAg-negative group. Fifty-two HBsAg-negative samples were sequenced, revealing high-frequency amino acid substitution sites in the HBV S protein, including immune escape mutations (i.e., Y100C, S114T, C124Y, P127L, G130R, T131N, M133T, C137S, G145A) and TMD region substitutions (i.e., E2K/R/D, G7D/R, G10D, A17R, F20L/S, L21V, L22V).

**Conclusions:**

According to the results of T-lymphocyte subsets and serum cytokines, it can be deduced that the cellular immune function of HBsAg-negative patients is superior to that of HBsAg-positive patients, with attenuation of liver inflammation. HBsAg-negative patients may show a variety of mutations and amino acid replacement sites at high frequency in the HBV S region, and these mutations may lead to undetectable HBsAg, HBsAg antigenic changes or secretion inhibition.

Hepatitis B virus (HBV) infection is a major global health problem. According to the latest data released by the WHO in 2020, there are approximately 257 million cases of chronic HBV infection worldwide. Moreover, approximately 887,000 people die each year due to uncompensated liver cirrhosis, liver failure, primary liver cancer and other end-stage liver diseases caused by HBV infection, indicating that the HBV infection rate has not decreased (1). In hepatitis B virus infection, the most representative serological marker is hepatitis B surface antigen (HBsAg), but some patients infected with hepatitis B virus infection may be negative for HBsAg. Occult HBV infection (OBI) is described as having HBV DNA in the blood, and the current detection methods do not include hepatitis B surface antigen (HBsAg) (2). Loss of HBsAg and anti-HBs seroconversion are considered signs of hepatitis B virus (HBV) elimination. However, serum/intrahepatic HBV DNA can be found in some patients who are negative for serum HBsAg. This status is described as OBI (1, 2). OBI may lead to progression of liver cirrhosis and eventually become an important risk factor for hepatocellular carcinoma (3). Cases of OBI arise from a variety of sources, including patients who are clinically cured of chronic hepatitis B, with HBsAg being serologically cleared but with a small amount of HBV remaining in the liver, or patients infected with mutant HBV (3–5). In addition, it is important to emphasize that the window of acute hepatitis B virus infection is not OBI patients. At present, the mechanism of OBI and how it participates in the occurrence of liver injury remain uncertain, and more studies and long-term clinical follow-up are necessary to better understand the viral and host biological mechanisms and clinical significance of OBI. This study included patients with HBsAg-negative and HBV DNA-positive states in the clinic.

There are several mechanisms that might explain such a state of infection, including the following: mutations in HBV genes, particularly HBsAg escape mutations that are undetectable by commercial HBsAg assays; host mechanisms that lead to strong suppression of HBV replication and transcription; host immune surveillance; coinfection with other viruses, such as hepatitis C virus (HCV); and epigenetic mechanisms (6). The HBV S region can be divided into three sections: N-terminal (aa 1-99), major hydrophilic region (MHR) (aa 100-169) and C-terminal (aa 170-226) regions (7, 8). The ‘α’ antigen determinant (aa 124-147 or 149) in the major hydrophilic region (MHR) is the primary target by which HBsAg is recognized by anti-HBs and immune cells (9). There are four transmembrane domains of HBsAg (TMDs): TMD1 (aa 4-24), TMD2 (aa 80-98), TMD3 (aa 160-193) and TMD4 (aa 202-222). Amino acid substitution in and around the ‘α’ antigen determinant may change the conformation of HBsAg and T-cell epitopes, altering immunogenicity (10). HBV S gene mutations are known to influence the occurrence of HBsAg-negative and HBV DNA-positive cases. These mutations are called immune escape-associated mutations, and they are mainly located in the MHR. A variety of mutations associated with HBsAg-negative and HBV DNA-positive cases have been discovered. The most well-known immune escape mutant is G145R, which impairs HBsAg secretion, antigenicity and anti-HBs binding (11).

It is generally believed that HBV infection can cause disruption of the function and proliferation of various immune cells involved in innate and adaptive immune responses. In general, the result of HBV infection is determined by the interaction between the virus and host (12). HBV itself is a noncytopathogenic virus, and liver damage is mainly attributed to the host's immune response. During HBV infection, the host immune response is a double-edged sword that defends against infection by destroying virus-infected cells while inducing liver inflammation and aggravating liver injury (13, 14). Cellular immunity is crucial in the occurrence and progression of hepatitis B disease. T helper cells (Th cells) are classified into Th1 and Th2 subsets according to the different cytokines produced, and the Th1/Th2 balance is critical for maintaining immune function. In chronic HBV infection, Th cells secrete high levels of Th2-type cytokines, inducing the cellular immune balance toward Th2 cells, high expression of HBsAg in serum and liver tissues, a decrease in the number of T lymphocyte subsets, significantly reduced cytokine production ability, and functional exhaustion (15–17).

At present, clinical research on HBsAg-negative and HBV DNA-positive patients is still lacking. Based on a large amount of clinical data, serum HBsAg clearance after nucleotide antiviral treatment or spontaneously may endure for more than 6 months in a small number of HBV-infected patients while serum HBV DNA remains present at a low level in serum. Nevertheless, the majority of HBV-infected patients remain positive for HBsAg after antiviral treatment, and HBV DNA can become negative or be present in serum to varying degrees. The aim of this study was to elucidate the characteristics of T lymphocyte subsets, serum cytokines and HBV S gene status in HBsAg-negative and HBV DNA-positive patients.

## Materials and methods

### Population studied

This is a cross-sectional study. Between May 2019 and May 2022, 279 patients who were HBsAg negative(<0.05 IU/mL, S/CO<1) and HBV DNA positive(≥10 IU/mL) were screened by hospital data systems. Only 52 of the 279 patients had persistent HBsAg-/HBV DNA+ serological status(>3 months). They had all been diagnosed with chronic hepatitis B in the past. 52 HBsAg+/HBV DNA+ patients who were diagnosed with chronic hepatitis B were matched by sex, age, and disease type. At the same time, clinical residual serum samples from 16 healthy people were collected as the control group. The criteria for healthy subjects were: healthy, no liver disease, no other diseases, and normal laboratory tests. The HBsAg-positive patient samples came from the patient's first blood draw after admission, before antiviral treatment. The diagnostic criteria are based on WHO guidelines for the prevention and treatment of chronic hepatitis B (2019 version) (12). The exclusion criteria were as follows: HBV infection window period; HCV infection not combined with other viral infections; alcoholic liver disease; streptocarpus; drug-induced liver injury; autoimmune diseases, hematological tumors and other serious genetic and metabolic diseases; use of immunosuppressants and hormones within the past three months; and post chemotherapy. The study was approved by the Medical Ethics Committee of Capital Medical University, Beijing Youan Hospital. Because the study used clinical residual serum samples, it was exempt from informed consent.

### Laboratory assays

Roche automatic immaterialize was routinely used to detect serum HBsAg, anti-HBs, HBeAg, anti-HBe and anti-HBc at the hospital clinical examination center. HBsAg titer<0.05 IU/mL and anti-HBs<10 IU/L, HBeAg S/CO<1, or anti-HBe and anti-HBc S/CO>1 was considered negative. Quantitative detection of serum HBV DNA was carried out by Abbott Real-Time fluorescence quantitative PCR. The lower limit of quantification was 10 IU/mL (34.1 HBV DNA copies/mL) in the 0.5 mL sample preparation protocol. The results of the assay were expressed as undetected, detectable but below the lower limit of quantification (i.e., HBV DNA detected but not quantifiable), and calculated results in IU/mL within the linear range of the assay. The Siemens Aptio automatic biochemical assembly pipeline was used to detect liver function indexes, such as serum alanine aminotransferase (ALT), aspartate aminotransferase (AST), total bilirubin (TBIL), and direct bilirubin (DBIL).

### Detection of peripheral blood T-lymphocyte subsets

Whole-blood samples were analyzed using a BECKMAN flow cytometer (Coulter, USA). Lymphocytes were analyzed using a gate set on a forward scatter vs. side scatter, and three-color flow cytometry was applied to combine CD3, CD4 and CD8. The peripheral blood T-lymphocyte subsets detected in each sample were analyzed using CellQuest software (Coulter, USA).

### Cytokine and chemokine profiles

We use MILLIPLEX MAP Human Cytokine/Chemokine/Growth Factor Panel A 48 Plex Premixed Magnetic Bead Panel Kit (Merck, USA) following the manufacturer’s recommendations and a Luminex MAGPIX Instrument System, as used for simultaneous quantification of any or all of the following analytes in human tissue/cell lysate and culture supernatant samples and serum or plasma samples: Soluble Cluster of Differentiation 40 Ligand (sCD40L), epidermal growth factor (EGF), Eotaxin, fibroblast growth factor 2 (FGF-2), Fms-related tyrosine kinase 3 ligand (FLT-3L), Fractalkine, granulocyte-stimulating factor(G-CSF), granulocyte macrophage colony-stimulating factor (GM-CSF), growth-regulated oncogene-α (GRO-α), interferon-α2 (IFN-α2), interferon-γ (IFN-γ), IL-1α, IL-1β, interleukin 1 receptor antagonist (IL-1RA), IL-2, IL-3, IL-4, IL-5, IL-6, IL-7, IL-8, IL-9, IL-10, IL-12 (p40), IL-12(p70), IL-13, IL-15, IL-17A, IL-17E/IL-25, IL-17F, IL-18, IL-22, IL-27, interferon gamma-induced protein 10 (IP-10), monocyte chemoattractant protein 1 (MCP-1/CCL2), MCP-3, macrophage-stimulating factor(M-CSF), macrophage-derived chemokine (MDC), monokine induced by IFN-γ (MIG/CXCL9), macrophage inflammatory protein 1α (MIP-1α/CCL3), MIP-1β/CCL4, platelet-derived growth factor AA (PDGF-AA), PDGF-AB/BB, regulated upon activation normal T-Cell expressed and presumably secreted (RANTES/CCL5), TGF-α, tumor necrosis factor α (TNF-α), TNF-β/lymphotoxin alpha (LTA), and vascular endothelial growth factor A (VEGF-A). Serum cytokine profiles were quantitatively measured in 52 HBsAg-negative and HBV DNA-positive patients, 52 HBsAg-positive patients and 16 healthy subjects. MILLIPLEX® products are based on Luminex xMAP technology. Cytokines with more than 50% missing data were not analyzed. Cytokine concentrations have an intra-assay coefficient of variation within 15%. As 4 of the 48 cytokines were not detectable in more than 50% of the samples (GM-CSF, IL-3, IL-7, IL-22), these four cytokines were not analyzed. A variety of cytokine levels are shown in the **Supplementary Materials**.

### Sequencing analysis of the HBV S region

HBV DNA was extracted from 1.0 ml of serum using the silica gel membrane centrifugal column method. First-round and second-round PCR were performed to amplify the HBV S region using PrimeSTAR MAX DNA polymerase. The primers used for both rounds of PCR were PF (5’-TTCCTGC TGGTGGCTCCAGTTC-3’, nt54-75) and PR (5’-TTCCGCAGTATGGATCGGCAG-3’, nt1258-1278), amplifying a 1224-bp fragment. PCR amplicons were assessed by 1% agarose gel electrophoresis (200 V for 20 min), and positive amplicons were purified. Both strands of purified amplification products were sequenced directly using ABI 3730xl DNA Analyzer. Homology evaluations were performed with the GenBank database using BLAST analysis at https://www.ncbi.nlm.nih.gov. Nucleic and amino acid sequences were analyzed using MEGA 7.0 software. HBV was genotyped based on the full sequence of the S gene using an online prediction tool (https://hbv.geno2pheno.org/index.php). Amino acid substitutions were determined by comparing specimen sequences to the genotype consensus sequence from the alignment of genotype sequences downloaded from https://www.ncbi.nlm.nih.gov. The immune escape mutation of the HBV S protein was determined by the Geno-2-pheno-hbv tool (https://hbv.geno2pheno.org/index.php).

### Statistical analysis

Statistical analysis was performed using SPSS 26.0. Single-factor ANOVA was employed for comparison of continuous variables following a normal distribution and the *SNK-q* test for comparisons between groups. A t test was used to compare two groups of continuous variables. The *Kruskal-Wallis H* test was used to compare continuous variables with a nonnormal distribution between groups, and the *Mann-Whitney U* test was used to compare the two groups. Two groups of classifying variables were compared using the *X* ^2^ test. Continuous variables are expressed as ( $\bar{x}$ ±s) or the median (P25-P75), and a difference was considered to be statistically significant at *P*< 0.05. GraphPad Prism 8 was utilized for the statistical analysis.

## Results

### Basic clinical data and laboratory test results

The HBsAg-negative patients were mainly male, at 76.92% (40/52), and the mean age was 53 years old. Most of the HBsAg-negative patients had received antiviral therapy with nucleotide analogs and/or interferon according to their clinical history. The positive rates of anti-HBs and anti-HBe in HBsAg-negative patients were significantly higher than those among HBsAg-positive patients[51.92%(27/52) vs. 0.00%(0/52), 80.77% (42/52) vs. 57.69%(30/52)] (*P*<0.05). A total of 80.77% of HBsAg-negative patients had HBV DNA below 200 IU/mL, and the median value of HBV DNA load was significantly lower than that of HBsAg-positive patients (log, 1.76 vs. 5.77 IU/mL) (*P*<0.05). Compared with HBsAg-positive patients, median levels of ALT, AST and TBA in HBsAg-negative patients were lower (36 vs. 103 U/L, 38 vs. 92 U/L and 12.40 vs. 120.00 μmol/L), and the differences were significant (*P*<0.05) (**Table 1**).

**Table 1:** General clinical information of HBsAg negative group, HBsAg positive group, and healthy control group.

	HBsAg negative group (N=52)	HBsAg positive group (N=52)	HC (N=16)	P values
Gender				
Female (%)	12 (23.08)	12 (23.08)	5 (31.25)	0.327
Male (%)	40 (76.92)	40 (76.92)	11 (68.75)	0.327
Age (years)	53±12^#^	51±12	37±4	0.01
HBsAg + (%)	0 (0)^*^	100 (100.00)	0 (0)	<0.001
Anti-HBs + (%)	27 (51.92)^*#^	0 (0)	16 (100.00)	<0.001
HBeAg + (%)	1 (1.92)^*#^	27 (42.31)	0 (0)	<0.001
Anti-HBe + (%)	42 (80.77)^*#^	30 (57.69)	0 (0)	<0.001
HBV DNA (IU/mL)				
<200 (%)	42 (80.77)^*#^	6 (11.54)	0 (0)	<0.001
≥200 (%)	10 (19.23)^*#^	46 (88.46)	0 (0)	<0.001
HBV DNA load (log, IU/mL)	1.76 (1.30-2.20)^*#^	3.58 (2.67-5.91)	0 (0)	<0.001
ALT (U/L)	36.00 (21.25-84.00)^*#^	103.00 (53.255-200.00)	15.50 (12.25-21.75)	<0.001
AST (U/L)	38.00 (22.25-102.00)^*#^	92.00 (44.50-175.00)	22.50 (18.00-24.75)	<0.001
TBIL (μmol/L)	25.60 (16.33-74.75)^#^	27.5 (17.35-85.73)	16.25 (12.23-17.90)	0.001
DBIL (μmol/L)	10.40 (6.35-52.40)^#^	12.75 (5.30-104.60)	4.40 (3.73-5.88)	<0.001
TP (g/L)	68.05 (62.15-73.40)	71.60 (62.28-76.65)	67.45 (60.28-77.03)	0.601
TBA (μmol/L)	12.40 (4.23-61.08)	120.00 (78.00-169.00)	19.55 (9.38-53.90)	<0.001
Antivirals-experienced (%)	43 (82.69)^*#^	0 (0)	0 (0)	<0.001

^*^, compared with HBsAg positive group, the difference was statistically significant(P<0.05); ^#^, compared with healthy control group(HC), the difference was statistically significant(P<0.05); P values, Compare the three groups; ALT, alanine aminotransferase. AST, aspartate aminotransferase. CHB, Chronic hepatitis B. LC, liver cirrhosis. HCC, Hepatocellular carcinoma. HBsAg-, HBsAg negative and HBV DNA positive. HBsAg+, HBsAg positive. HC, Healthy control. ^α^Antiviral experience means that patients who have been treated with nucleotide analogues and/or peg-interferon-α2b can be found in their medical records (Those patients are still receiving the antivirals treatment).

### Characteristics of peripheral blood T-lymphocyte subsets

**Figure 1:**
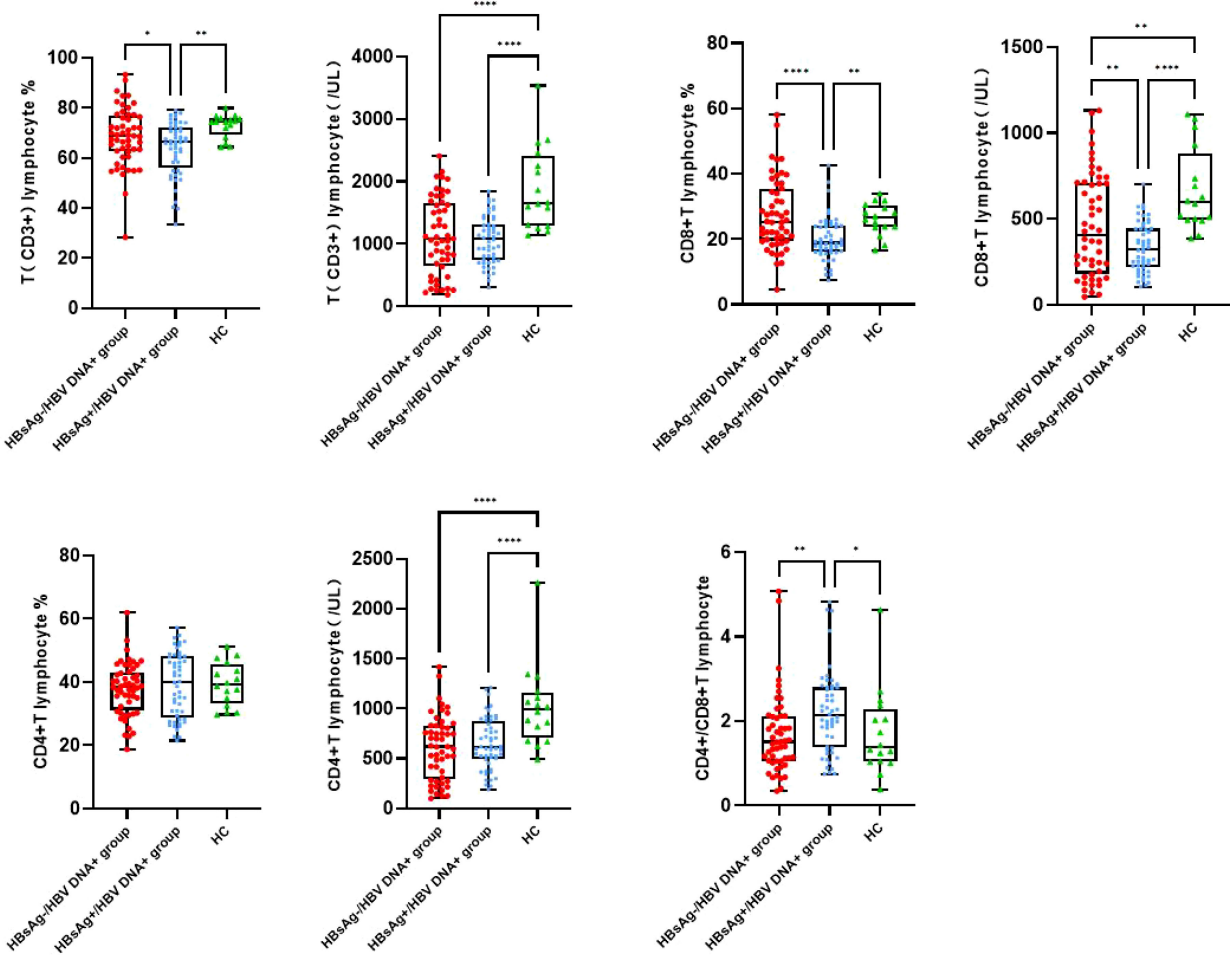
Peripheral blood T lymphocyte subsets among the three groups. Comparison of the mean of T lymphocyte, CD4 and CD8 T lymphocyte and CD4/CD8 ratio. HBsAg-, HBsAg negative and HBV DNA positive. HBsAg+, HBsAg positive. *P<0.05, **P<0.01, ****P<0.0001.

The number of T lymphocytes and CD8+T lymphocytes decreased successively in the healthy control group, HBsAg-negative group and HBsAg-positive group. Each group of data was compared with the mean and median(**Table 2**). Levels of T lymphocytes and CD8+ T lymphocytes in HBsAg-negative group were higher than HBsAg-positive group, and the difference was statistically significant (*P*<0.05). The number of T-lymphocyte subsets in the HBsAg negative group and HBsAg positive group was lower than that in the healthy control group. Ratios of T lymphocytes/lymphocytes(T lymphocyte%), T lymphocytes, CD8+T lymphocytes/ lymphocytes(CD8+T lymphocyte%) and CD8+T lymphocytes in the HBsAg-negative group were significantly lower than those in the HBsAg-positive group(*P*<0.05). However, the CD4/CD8 ratio was the highest in HBsAg-positive group, and the difference was statistically significant compared with the other two groups(*P*<0.05). There was no difference in the CD4/CD8 ratio between HBsAg-negative group and healthy control group (**Figure 1**).

**Table 2:** Comparison of T lymphocyte subsets among three groups.

Cytokines (pg/mL)	HBsAg- group(N=52)	HBsAg+ group(N=52)	HC(N=16)	*P* values
	Median(P25-P75) $\bar{X}$ ±SD	Median(P25-P75) $\bar{X}$ ±SD	Median(P25-P75) $\bar{X}$ ±SD	
T lymphocyte %	69.02 (62.60-77.08)^*#^	66.25 (55.88-72.21)	74.45 (69.18-75.85)	<0.001
	68.70±11.79^*^	63.73±10.81	72.92±4.71	<0.001
T lymphocyte (/μL)	1077 (650-1661)^#^	1086 (737-1312)	1654 (1289-2406)	<0.001
	1129±610^#^	1061±376	1889±673	<0.001
CD8 T lymphocyte %	25.31 (19.57-35.29)^*^	18.86 (16.16-24.07)	26.55 (23.65-30.34)	<0.001
	27.49±10.97^*^	20.09±6.74	26.20±4.93	<0.001
CD8 T lymphocyte (/μL)	405 (181-710)^*#^	321 (217-443)	598 (496-882)	<0.001
	459±301^*#^	332±145	669±242	<0.001
CD4 T lymphocyte %	38.55 (31.14-43.13)	40.03 (28.62-48.18)	39.29 (33.15-45.60)	0.329
	37.86±8.29	39.08±10.26	39.50±6.82	0.575
CD4 T lymphocyte (/μL)	619 (291-826)^#^	609 (494-874)	994 (712-1159)	<0.001
	607±320^#^	649±258	1021±412	<0.001
CD4/CD8	1.51 (1.05-2.12)	2.13 (1.37-2.80)^*^	1.38 (1.04-2.29)^#^	0.017
	1.68±0.94	2.21±0.98	1.72±1.02	0.005

^*^, compared with HBsAg positive group, the difference was statistically significant(P<0.05); ^#^, compared with healthy control group(HC), the difference was statistically significant(P<0.05); P values, Compare the three groups; T lymphocyte %, T lymphocyte/lymphocyte; CD8 T lymphocyte %, CD8 T lymphocyte/lymphocyte; CD4 T lymphocyte%, CD4 T lymphocyte/lymphocyte; The data is represented by the median(P25-P75).

### Levels of serum cytokines and chemokines

**Figure 2:**
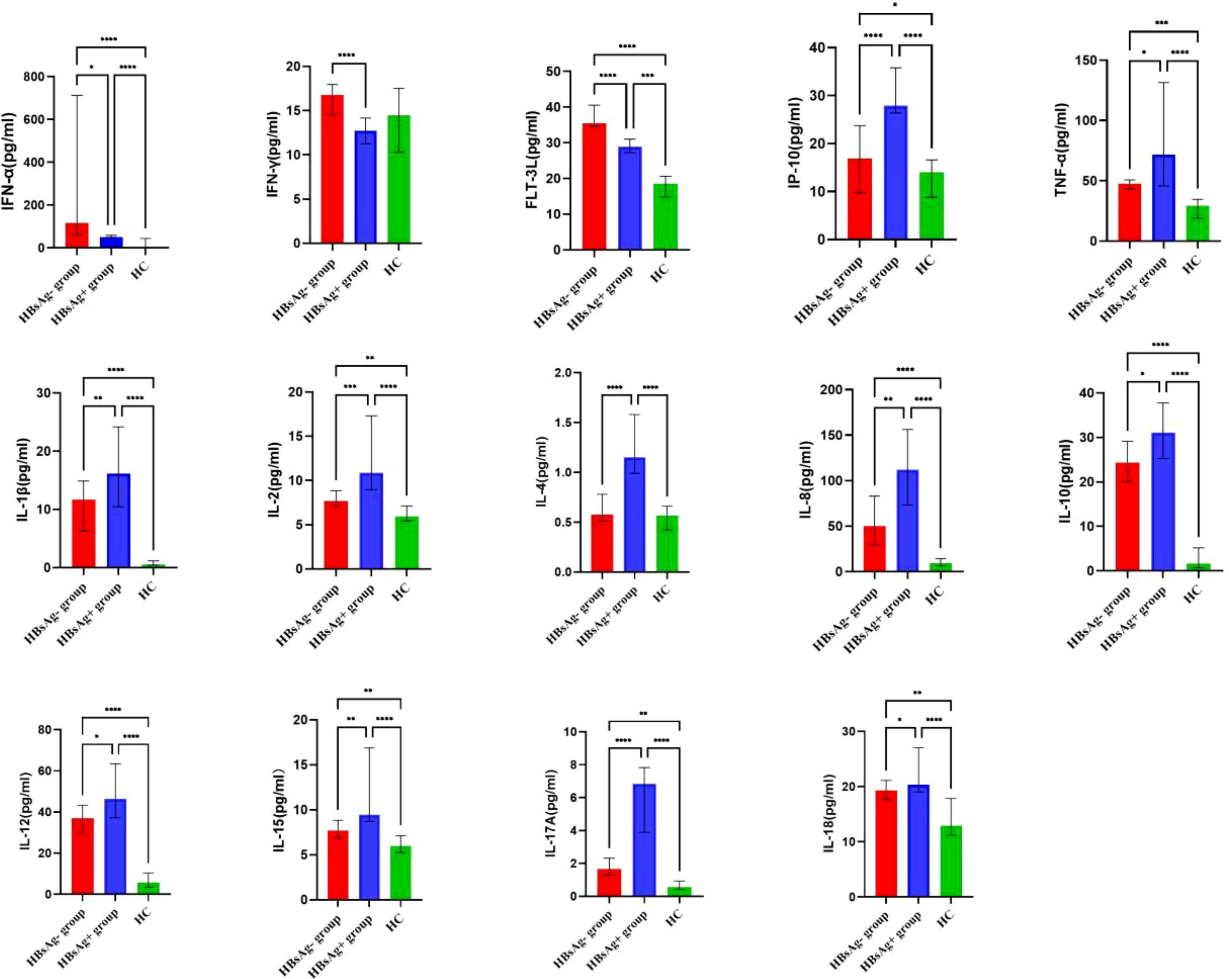
Comparison of the median levels of serum cytokine in the HBsAg negative, HBsAg positive and healthy control group. The figure shows the median with 95%CI of cytokines in three groups patients. *Kruskal-Wallis H* test was used for comparison among the three groups, and *Benjamini* test was used for pairwise comparison between groups. HBsAg-, HBsAg negative and HBV DNA positive. HBsAg+, HBsAg positive. *P<0.05. **P<0.01. ***P<0.001, ****P<0.0001.

By comparing median levels of cytokines and chemokines between the HBsAg-negative group and HBsAg-positive groups, significant differences in IFN-γ, IFN-α, FLT-3L, IP-10, TNF-α, IL-1β, IL-2, IL-4, IL-8, IL-10, IL-12, IL-15 IL-17A and IL-18 were found (*P*<0.05). Additionally, higher median values of IFN-γ, IFN-α and FLT-3L were detected in the HBsAg-negative group and higher median levels of IL-1β, IL-2, IL-4, IL-8, IL-10, IL-12, IL-15, IL-17A and IL-18 were detected in the HBsAg-positive group (**Figure 2**, **Table 3**). Among the 48 cytokines and chemokines detected, 14 each showed significant differences between the two groups, with the other cytokines having no significant difference. Furthermore, median levels of IFN-γ and FLT-3 L in the HBsAg-positive group were lower than those in the HBsAg-negative group and healthy controls, with significant differences (*P*<0.05); median values of IFN-α were highest in the HBsAg-negative group, with significant differences between groups (*P*<0.05). Moreover, median levels of IFN-α, IP-10, TNF-α, IL-1β, IL-2, IL-8, IL-10, IL-12, IL-15, IL-17A and IL-18 were higher in the HBsAg-negative and HBsAg-positive groups than in the healthy controls, a statistically significant difference (*P*<0.05). The median (P25-P75) and differences in cytokines in the three groups are shown in **Figure 2** and **Table 3**.

**Table 3:** Circulating cytokine profiles in HBsAg negative, HBsAg positive, and Healthy control group.

Cytokines (pg/mL)	HBsAg- group(N=52)	HBsAg+ group(N=52)	HC(N=16)	*P* values
	Median(P25-P75) $\bar{X}$ ±SD	Median(P25-P75) $\bar{X}$ ±SD	Median(P25-P75) $\bar{X}$ ±SD	
IFN-γ	16.74 (13.01-19.38)^*^	12.74 (10.45-14.54)	14.46 (10.53-17.40)	<0.001
	19.84±13.31	13.17±3.41	14.40±3.68	
IFN-α	116.90 (40.74-784.2)^*#^	49.47 (36.56-274.50)	0.94 (0.52-34.01)	<0.001
	419.90±459.90	290.00±497.20	16.65±31.76	
FLT-3L	35.45 (32.67-42.04)^*#^	29.97 (26.79-40.77)	18.94 (15.99-21.02)	<0.001
	38.00±7.68	36.77±18.42	19.43±8.34	
TNF-α	47.73 (38.88-55.56)^*#^	71.51 (42.47-160.40)	29.24 (19.31-34.09)	<0.001
	53.11±26.68	136.00±161.90	30.00±11.44	
IP-10	33.30 (19.54-61.67)^*#^	24.31 (16.23-34.11)	54.06 (40.33-72.08)	<0.001
	49.56±48.54	29.41±21.13	56.51±20.59	
IL-1β	11.66 (2.95-21.61)^*#^	16.18 (7.71-74.11)	0.58 (0.47-1.11)	<0.001
	16.14±14.77	73.16±121.20	2.05±4.02	
IL-2	7.68 (6.77-9.43)^*#^	10.85 (8.20-19.21)	5.95 (5.48-6.95)	<0.001
	9.84±6.06	15.37±9.31	6.20±1.01	
IL-4	0.58 (0.49-1.09)^*^	1.15 (0.81-2.65)	0.57 (0.45-0.66)	<0.001
	0.78±0.41	1.59±1.10	0.57±0.13	
IL-8	49.92 (21.49-126.70)^*#^	111.80 (55.99-273.60)	9.32 (6.71-13.62)	<0.001
	120.90±193,40	191.70±220.10	11.00±6.04	
IL-10	24.34 (15.77-40.67)^*#^	31.06 (21.88-66.33)	1.58 (0.70-5.18)	<0.001
	31.93±26.00	53.75±48.69	3.18±3.47	
IL-12 (p40)	36.94 (27.18-52.58)^*#^	46.36 (31.50-89.73)	5.64 (3.63-9.49)	<0.001
	39.59±19.86	62.70±40.58	6.53±4.28	
IL-15	7.68 (6.52-9.43)^*#^	9.43 (7.45-18.96)	5.95 (5.38-6.64)	<0.001
	9.79±6.09	14.03±8.72	6.07±0.89	
IL-17A	1.67 (1.23-3.04)^*#^	6.81 (3.08-10.67)	0.55 (0.42-0.87)	<0.001
	2.71±3.59	8.11±7.00	3.15±9.15	
IL-18	19.31 (16.41-22.19)^*#^	20.32 (18-28.17)	12.94 (11.35-17.49)	<0.001
	19.94±7.70	25.63±14.16	14.03±4.39	

SD, standard deviation. P25, 25th percentile. P75, 75th percentile. HBsAg-, HBsAg negative and HBV DNA positive. HBsAg+, HBsAg positive. HC, healthy control group. To know the difference between the groups, the Mann–Whitney U nonparametric test was used.: ^*^, compared with HBsAg positive group, the difference was statistically significant(P<0.05); ^#^, compared with healthy control group(HC), the difference was statistically significant(P<0.05); P values, Compare the three groups.

### HBV S region mutation and amino acid substitution in HBsAg-negative and HBV DNA-positive patients

Only 43 of 52 HBsAg-negative samples were successfully sequenced; thus, 43 HBV S gene sequences were analyzed. Genotypes of HBV were determined according to the S gene sequence. Of the 30 HBV S region fragments identified in HBsAg-negative strains, 30 were classified as genotype B (30/43, 69.77%) and 13 strains as genotype C (13/43, 31.23%). DNA sequencing revealed an amino acid substitution in the HBV S protein in 39 of the 43 (90.70%) HBsAg-negative and HBV DNA-positive patients. E kinds of immune escape mutation sites were detected in the genotype B HBV S protein, namely, G119D, T126I, T127P, A128V, G130R, S132F, M133K and C137S, in six HBsAg-negative and HBV DNA-positive patients (6/43, 13.95%). Additionally, 8 kinds of immune escape mutation sites were detected in the genotype C HBV S protein in five HBsAg-negative and HBV DNA-positive patients: Y100C, S114T, C124Y, P127L, G130R, T131N, M133T and G145A (5/43, 11.63%). Amino acid substitution in the transmembrane domain (TMD) region was observed in 31 HBsAg-negative patients (31/43, 72.09%), but only 8 HBsAg-negative samples showed an amino acid substitution in the "α" determinant (amino acids 124-147) of the S protein (**Table 4**). One female patient’s HBV DNA load was as high as 7.81×10^5^ IU/mL, but HBsAg was negative after antiviral treatment with entecavir. In her sample, we detected many HBV S region mutations resulting in multiple amino acid replacement sites, including immune escape mutations (S114T, C124Y, P127L, G130R, T131N and M133T) and TMD region mutations (L8H, V14A, F19S, V168A, S174N, L175S, Q181R and P203R). There were also three patients with high HBV DNA loads at more than 1×10^4^ IU/mL, and they also showed many HBV S region mutations (**Table 4**).

**Table 4:** HBsAg negative patients with amino acids substitutions in HBV S protein.

Sample	Sex	HBV DNA oading(IU/mL)	Anti-HBc	Anti-HBs level(IU/L)	HBV genotype	S protein amino acid substitutions	Immune escape mutations	TMD mutations
1	F	73	Pos	34.9	B	N40S, L49H, F200Y, S204N, V224A	N	S204N
2	F	7.81×10^5^	Pos	12.6	C	L8H, V14A, F19S, I57T, I68T, S114T, C124Y, P127L, G130R, T131N, M133T, V168A, S174N, L175S, Q181R, P203R, M213I	S114T, C124Y, P127L, G130R, T131N, M133T	L8H, V14A, F19S, V168A, S174N, L175S, Q181R, P203R
3	M	196	Pos	844	B	F219V	N	F219V
4	M	159	Pos	25.4	B	N207H	N	N207H
5	M	117	Pos	61.9	B	E2D	N	N
6	M	136	Pos	neg	C	V18G, Y100C, V159A, I218L	Y100C	V18G, I218L
7	M	62	Pos	neg	B	E2R, L13I, F20L, L22V, T23D, G50A	N	L13I, F20L, L22V
8	M	307	Pos	neg	C	Y100C, I218L	Y100C	I218L
9	F	110	Pos	neg	B	E2K, L22V	N	L22V
10	F	31	Pos	955.2	B	P135S	N	N
11	M	96	Pos	252.8	B	L12C, L13R, A17P, F19L, G50A	N	L12C, L13R, A17P, F19L
12	M	39.9	Pos	184	B	L21C, L22V	N	L21C
13	M	21.7	Pos	neg	B	N40Q, R78G	N	N
14	M	4853	Pos	neg	B	S132F, C137S	S132F, C137S	N
15	F	215	Pos	neg	B	T118P, T125P, T126I, A128V, G130R, S132F, M133K, C137S	T126I, A128V, G130R, S132F, M133K, C137S	
16	M	178	Pos	110.4	B	F20S	N	F20S
17	M	747	Pos	neg	B	F93S, L95W. V96S. V106L C107S. PIOSL, G119D	G119D	F93S, L95W. V96S
18	M	155	Pos	82.61	B	C48S. P66L. P70L F80L I86F. 192N. F93L, L94C. L95G. V96F. C107V. PLOSS	N	I86F. 192N. F93L. L94C, L95G. V96F
19	M	28	Pos	20.20	B	F20L, L2IV, T27P. I28N, P29T	N	F20L. L21V
20	F	73	Pos	19.5	B	N40S. L49H, F200Y, S204N. V224A	N	F200Y, S204N
21	M	<10	Pos	neg	B	F20L, L2IV, L22V	N	F20L. L21V. L22V
22	M	45	Pos	1000	C	L15V. I37S, L39V. N40R F41V. L42P	N	L15V
23	M	20	Pos	neg	C	N	N	N
24	M	48	Pos	neg	C	V14G L15V, P46L. T47K I68T	N	V14G,L15V
25	M	45.3	Pos	neg	C	N31 P49L ISST. L98V. V184A	N	L98V. V184A
26	M	146	Pos	neg	C	GIOR G145A L216F	G145A	N
27	M	814	Pos	1000	B	W35C. L39P. S58C, R79H, T127P. W182*	T127P	W182*
28	F	41.6	Pos	neg	C	C65G. G71C, V184A	N	V184A
29	M	20	Pos	neg	C	N	N	N
30	M	104	Pos	1000	C	F19V. L22V. T23D. S38L	N	F19V. L22V. T23D
31	M	773	Pos	neg	C	GIOR G145A R160G W201G P217Q. D28F	G145A	W201G P217Q. 1218F
32	F	23.8	Pos	neg	C	N	N	N
33	M	22	Pos	46.47	B	G7R L9*. GIOD. F20L T27P	N	G7R L9*. G10D. F20L
34	M	100	Pos	neg	B	F20L	N	F20L
35	M	20	Pos	52.48	B	N	N	N
36	M	104	Pos	1000	B	G7R GIOD. A17R GISV. F20L L21V	N	G7R G10D,A17R G18V. F20L, L21V
37	M	595	Pos	187.8	B	G10D. A17R	N	G10D. A17R
38	F	110	Pos	1000	B	G10D. F20S. T27P	N	G10D, F20S
39	F	20	Pos	362.7	B	Q5IK N52L C64L C65S. 168T. C69R G71A FSOL L97F. L98W	N	L97F. L98W
40	M	53	Pos	neg	B	G7D. A17R F20L L21V,L22V	N	G7R, A17R, F20L. L21V. L22V
41	F	1097	Pos	1000	B	M133K C137S	M133K C137S	N
42	M	1.28*10	Pos	neg	B	T125P. A128V, S132E	A128V. S132F	N
43	M	20	Pos	neg	B	G10D. L22* T23Q	N	GIOD. L22*. T23Q

F, Female. M, Male. Pos, positive. neg, negtive. N, none. TMD, transmembrane domain. *, stop codon.

### Amino acid substitution frequency in HBV S protein

A total of 43 samples from HBsAg-negative patients and 49 samples from HBsAg-positive patients were successfully sequenced. Among 43 HBV S-region sequences in HBsAg-negative patients, 30 (30/43, 69.77%) were genotype B and 13 (13/43, 31.23%) genotype C; 34 of 49 (34/49, 69.39%) HBV S-region sequences in HBsAg-positive patients were genotype B and 15 (15/49, 31.23%) genotype C. The mutation rate of the HBV S protein in HBsAg-negative patients was 90.70% (39/43) and that in HBsAg-positive patients was 28.57% (14/49), significantly higher in HBsAg-negative patients. F20L/S (9/30, 30.00%) displayed the highest occurrence frequency in HBsAg-negative patients with genotype B, followed by L22V (5/30, 16.67%) and G10D (5/30, 16.67%). The frequency of multiple immune escape mutations in HBsAg-negative patients with the B and C genotypes was higher than that in HBsAg-positive patients. A variety of amino acid substitution sites also occurred at high frequency in the TMD region of the S protein in HBsAg-negative patients. The details of amino acid substitution frequencies for HBsAg-negative and HBsAg-positive patients are provided in **Table 5**.

**Table 5:** Frequency of amino acid substitution of HBV S protein in HBsAg negative and HBsAg positive patients.

Amino acid substitution site(%)	Genotype B			Amino acid substitution site(%)	Genotype C		
	HBsAg- group (N=30)	HBsAg+ group (N=34)	*P* values		HBsAg- group (N=13)	HBsAg+ group (N=15)	*P* values
E2K/R/D	3 (10.00)	0 (0.00)*	0.001	N3I	1 (7.69)	0 (0.00)*	0.004
G7D/R	3 (10.00)	0 (0.00)*	0.001	V14G/A	2 (15.38)	0 (0.00)*	<0.001
G10D	5 (16.67)	1 (2.94)*	0.001	G10R	2 (15.38)	1 (6.67)	0.071
A17R	3 (10.00)	0 (0.00)*	0.001	F19V/S	3 (23.08)	0 (0.00)*	<0.001
F20L/S	9 (30.00)	1 (2.94)*	<0.001	L15V	2 (15.38)	0 (0.00)*	<0.001
L21V	4 (13.33)	1 (2.94)*	0.009	L22V	1 (7.69)	0 (0.00)*	0.004
L22V	5 (16.67)	0 (0.00)*	<0.001	T23D	2 (15.38)	0 (0.00)*	<0.001
T27P	3 (10.00)	0 (0.00)*	0.001	I68T	3 (23.08)	2 (13.33)	0.066
G50A	2 (6.67)	0 (0.00)*	0.007	L98V	1 (7.69)	0 (0.00)*	0.004
T125P	2 (4.65)	0 (0.00)*	0.007	Y100C	2 (15.38)	0 (0.00)*	<0.001
T127P	2 (4.65)	0 (0.00)*	0.007	S114T	1 (7.69)	0 (0.00)*	0.004
A128V	2 (4.65)	0 (0.00)*	0.007	C124Y	1 (7.69)	0 (0.00)*	0.004
S132F	3 (6.98)	0 (0.00)*	0.001	P127L	1 (7.69)	0 (0.00)*	0.004
M133K	2 (4.65)	0 (0.00)*	0.007	G130R	1 (7.69)	0 (0.00)*	0.004
C137S	3 (6.98)	0 (0.00)*	0.001	T131N	1 (7.69)	0 (0.00)*	0.004
F200Y	2 (4.65)	0 (0.00)*	0.007	M133T	1 (7.69)	0 (0.00)*	0.004
S204N	2 (4.65)	0 (0.00)*	0.007	G145A	2 (15.38)	0 (0.00)*	<0.001
V224A	2 (4.65)	0 (0.00)*	0.007	V159A	1 (7.69)	0 (0.00)*	0.004
				V184A	2 (15.38)	0 (0.00)*	<0.001
				I218L	2 (15.38)	0 (0.00)*	<0.001

*, compared with HBsAg- group, the difference was statistically significant(P<0.05).

## Discussion

In this study, HBsAg-negative patients were treated with antiviral therapy or spontaneously experienced a gradual decrease in HBsAg titer until it disappeared; the HBV DNA load also decreased but remained at a low level in serum. This paper describes T-lymphocyte subsets, various cytokine levels and HBV S gene mutations in HBsAg-negative and HBV DNA-positive patients. The subjects of this study were clinically HBsAg-negative and HBV DNA-positive patients, and their serological characteristics and viral load were the same as those of OBI patients among blood donors in previous studies (18–20). Hence, regardless of whether HBsAg-negative cases are caused by antiviral therapy or naturally occurring OBI, clinical indicator characteristics are the same.

HBeAg is a marker of active HBV DNA replication in hepatocytes. However, most HBsAg-negative patients are negative for HBeAg, suggesting low viral replication in these patients. The positive rates of anti-HBs and anti-HBe in HBsAg-negative and HBV DNA-positive patients in our study were 51.92% and 80.77%, respectively, significantly higher than those in HBsAg-positive patients. Thus, most HBsAg-negative patients have undergone serological conversion, with low levels of viral replication and protein expression. To explore the host immune function and inflammatory state in HBsAg-negative patients, levels of T lymphocyte subsets and serum cytokines were analyzed in this study; the HBV S gene was also sequenced and analyzed. Despite suppression of HBV DNA replication and protein expression, the virus may not be completely eliminated. In fact, a small amount of HBV cccDNA remains in liver tissue, with the potential to progress to cirrhosis and hepatocellular carcinoma. As it is considered a risk factor for hepatocellular carcinoma, it cannot be ignored (21–23).

Some studies have suggested that HBsAg-negative and HBV DNA-positive cases are related to viral genome mutations, that is, HBsAg antigenicity changes in HBsAg-negative and HBV DNA-positive patients with mutations in the HBV PreS/S region. Mutations located in the immunodominant "α" determinant region (aa124-147 or 149) can impair the efficiency of HBsAg detection reagents by reducing the binding affinity of the HBsAg capture antibody (24). A single mutation in the "α" determinant in the main hydrophilic region (MHR) of the HBV S region can also inhibit HBsAg secretion (25). Additionally, amino acid substitution in the reverse transcriptase (RT) domain may lead to low levels of HBV DNA replication and HBsAg synthesis, resulting in HBsAg-negative and HBV DNA-positive cases (26). The substitutions M133T and T131N generate an extra N-linked glycosylation site that reportedly does not affect HBsAg secretion but may mask antigenic sites affecting detection (27). Moreover, the mutants Y100C and P127L may affect HBsAg expression and secretion as well as anti-HBs binding (28).

Among the 43 successfully sequenced HBsAg-negative samples, 39 HBV S region fragments showed mutations; among HBsAg-positive samples, 49 samples were sequenced successfully, and 14 HBV S region fragments showed mutations. The mutation rate of the HBV S gene in HBsAg-negative patients (39/43, 90.70%) was significantly higher than that in HBsAg-positive patients (14/49, 28.57%) in our study. Six HBsAg-negative samples showed immune escape mutation sites in the genotype B HBV S region, G119D, T126I, T127P, A128V, G130R, S132F, M133K and C137S (29), with immune escape mutation sites in the genotype C HBV S region in five HBsAg-negative samples, Y100C, S114T, C124Y, P127L, G130R, T131N, M133T and G145A (29). No immune escape mutation was detected in HBsAg-positive patients. These HBsAg immune escape mutants may have arisen as a result of specific selection, such as through the host immune system due to vaccination or antiviral selective pressure attenuating production of HBsAg and resulting in low plasma HBV DNA levels (30). Furthermore, these mutations may be responsible for virus detection failure in routine screening (25). Indeed, this is common in the clinical setting in which patients may have HBsAg-negative results but classical symptoms and, if tested for HBV DNA, will show high HBV DNA levels. In general, detection of some of these emerging mutants has become a major challenge for commercially available immunoassays. Thirty-one HBsAg-negative samples in the TMD region exhibited multiple amino acid substitution sites, many of which were newly discovered, such as E2K/R/D, G10D, A17R, F20L/S, L21V, L22V, F200Y, and S204N. E2 site mutations in the S protein are confirmed to impair secretion of HBsAg, which significantly affected detection of HBsAg (31). For genotype B, the highest mutation frequency of the S protein in HBsAg-negative patients was sF20L/S; regarding genotype C, sF19V/S had the highest frequency. Both mutations occur in the TMD region of the S protein. sF20L/S and sF19V/S are high-frequency mutations associated with HBsAg negativity, which may be a result of the pressure of antiviral therapy and have certain effects on HBsAg production or secretion. The truncation mutant sW182* was present in one HBsAg-negative patient in the study of Pollicino et al (32). This mutation induces retention of the truncated S protein in the perinuclear endoplasmic reticulum (ER) and is associated with lower HBV transcript levels due to decreased stability (33). Large-scale studies and *in vitro* experiments are needed to determine the significance of newly detected mutations.

Studies have shown that the immune system of HBsAg-negative and HBV DNA-positive patients strongly inhibits viral replication and gene expression (34–37). The ability of CD8 T lymphocytes to proliferate and secrete cytokines (e.g., IFN-γ, IL-2) is weakened in chronic HBV infection (38), and T-lymphocyte subsets can be used to assess the immune function of the host. As cytokines can alter the process of infection and affect the tendency and progression of chronic hepatitis B, they are worthy of study.

In this study, the number of CD4+ T lymphocytes, CD8+ T lymphocytes, CD4+ T lymphocytes/lymphocytes (CD4+ T%) and CD8+ T lymphocytes/lymphocytes (CD8+ T%) was lowest in the HBsAg-positive group, which may be related to the low immune function caused by chronic HBV infection. The numbers of CD4+ T cells and CD8+ T cells in the HBsAg-negative group were higher than those in the HBsAg-positive group. Levels of a variety of cytokines and chemokines (IFN-γ, IFN-α, FLT-3 L) related to immune regulation and virus clearance were also higher in the HBsAg-negative group. Therefore, the immune function of HBsAg-negative patients is enhanced compared with HBsAg-positive patients, which may be related to virus suppression in the former after antiviral treatment. The immune function of HBV-infected patients treated with antiviral interferon may also be enhanced, which can increase the clearance rate of HBsAg. Because this study was a cross-sectional study and we could only determine T lymphocyte subsets and serum cytokine levels after HBsAg clearance, it is unclear whether enhanced host immune function occurred before or after HBsAg clearance. Regardless, it can be confirmed that HBsAg-negative patients have strong host immune function. Although the number of CD4 and CD8 T cells in the HBsAg-negative patients was higher than that in the HBsAg-positive patients, it was lower than that in the healthy controls. Therefore, the immune function of HBsAg-negative patients did not completely recover to the normal level, which corresponds to the low level of serum HBV DNA in HBsAg-negative patients. A high level of HBsAg is associated with impairment of anti-HBV-specific T and B-cell immune function. Reducing the HBsAg level should promote recovery of specific immune function and in turn promote clearance of HBsAg (39, 40). In this study, we only analyzed the increase in overall T-lymphocyte subsets in HBsAg-negative patients and did not detect the immune function of HBV-specific T lymphocytes. Further research is needed.

Cytokines represent a large family of molecules, including the following: Th1-associated cytokines [e.g., IL-2 and IFN-γ], which have a functional contribution to cellular immune responses; Th2-associated cytokines (e.g., IL-4, IL-6, and IL-10), which have roles in humoral immune responses; regulatory T-cell (Treg)-associated cytokines [e.g., tumor growth factor-beta (TGF-β) and IL-10], which have been associated with immunomodulation and immunosuppression; and Th17-associated cytokines (e.g., IL-17, IL-22, and IL-23), which play critical roles in mediating inflammation (41). There are few studies on the production profile of cytokines and chemokines in HBsAg-negative and HBV DNA-positive patients, and the mechanism of liver injury is still unclear, but some studies have shown that persistence and transcription of HBV cccDNA in hepatocytes can stimulate production of cytokines, such as TNF-α and IFN-γ, resulting in hepatocyte injury (17). We conclude that levels of TNF-α and IFN-γ in HBsAg-negative patients are significantly higher than those in healthy subjects, which may be related to a small amount of HBV in the body. The persistence and transcription of HBV cccDNA in hepatocytes can lead to the production of cytokines, such as TNF-α and INF-γ (38). Cytokines and chemokines are essential effector molecules in the HBV-related inflammatory response. CD4+ T cells, CD8+ T cells, NK cells, DC cells and their related cytokines participate in the immune injury process of chronic HBV infection (42, 43).

In addition, IFN-γ, IFN-α and FLT-3 L levels in HBsAg-negative patients were significantly higher than those in HBsAg-positive patients in our study. High HBV DNA load and high HBeAg and HBsAg levels may inhibit immune cell function, leading to a reduction in FLT-3L, IFN-γ, and other cytokines with virus clearance effects and to an increase in the level of the most important cytokine for immunosuppression: IL-10 (43–45). HBsAg induces depletion phenotypes and dysfunction of T and B cells, leading to innate and adaptive immune deficiencies, and lowering serum HBsAg contributes to recovery of the host immune response (46). The increase in IFN-γ and FLT-3 L in the HBsAg-negative patients in the present study may be due to the decrease in HBV DNA and clearance of HBsAg after the use of nucleotide analog antiviral drugs. HBV DNA reduction and HBsAg clearance in HBsAg-negative patients after antiviral therapy may promote recovery of cellular immune function, thus leading to increased serum IFN-γ and FLT-3L levels. In general, levels of various proinflammatory cytokines (IL-2, IL-4, IL-8, IL-10, IL-12, IL-15, IL17A, IL-18) in HBsAg-positive patients were higher than those in HBsAg-negative patients and healthy people. Compared with HBsAg-positive patients, the inflammatory reaction of HBsAg-negative patients was reduced and the immune response function enhanced, but they were still in a state of HBV infection. Overall, a mild inflammatory response compared to healthy people was observed.

## Conclusion

High HBsAg serum levels lead to failure of the host immune system, preventing an effective antiviral response. Levels of HBsAg and virus decrease after antiviral treatment, and the inhibitory effect on host cellular immune function is weakened. Cellular immune function is gradually enhanced, which further increases the virus clearance effect. However, HBV S gene mutation may occur in HBsAg-negative patients during antiviral therapy, which leads to amino acid substitutions in the S protein. If the mutation occurs in the main hydrophilic region, the antigenicity of the surface antigen would be changed, rendering commercial detection reagents unable to detect it. If the mutation occurs in the TMD region and changes the S protein conformation, it may affect production and secretion of HBsAg and result in a concentration of serum HBsAg lower than the limit of detection. The decrease in HBsAg secretion is beneficial to host cellular immune function, and antiviral immune responses are continuously stimulated by persistent/intermittent low levels of HBV antigens. This study found a variety of new high-frequency mutation sites in clinically HBsAg-negative and HBV DNA-positive patients.

## Data availability statement

The original contributions presented in the study are publicly available. This data can be found here: https://doi.org/10.6084/m9.figshare.23903928.v1.

## Ethics statement

The study was approved by the Medical Ethics Committee of Capital Medical University, Beijing Youan Hospital. Because the study used clinical residual serum samples, it was exempt from informed consent.

## Author contributions

XL and S-XC are joint first authors(Equal & First authors). XL and S-XC are responsible for designing research plans, collecting clinical data, analyzing data, and writing first drafts of papers. HL participate in the review and editing of the first draft. J-LL participates in the acquisition of research funds and the review and revision of papers. All authors contributed to the article and approved the submitted version.

## Glossary

**Table:**

HBV	Hepatitis B virus
HBsAg negative and HBV DNA positive patients	Occult HBV infection
HBsAg	Hepatitis B virus surface antigen
anti-HBs	Hepatitis B virus surface antibody
HBeAg	Hepatitis B virus e antigen
anti-HBe	Hepatitis B virus e antibody
anti-HBc	Hepatitis B virus core antibody
ALT	alanine aminotransferase
AST	aspartate aminotransferase
TBIL	total bilirubin
DBIL	Indirect bilirubin
Alb	albumin
Glb	globulin
γ-GT	γ-glutamyl transpeptidase
ALP	alkaline phosphatase
CHB	Chronic hepatitis B
LC	liver cirrhosis
HCC	Hepatocellular carcinoma
PBMCs	peripheral blood mononuclear cells
sCD40L	Soluble Cluster of Differentiation 40 Ligand
EGF	epidermal growth factor
FGF-2	fibroblast growth factor 2
FLT-3L	Fms-related tyrosine kinase 3 ligand
G-CSF	granulocyte-stimulating factor
GM-CSF	granulocyte macrophage colony-stimulating factor
GRO-α	growth-regulated oncogene-α
IFN-α2	interferon-α2
IFN-γ	interferon-γ
IP-10	interferon gamma- induced protein 10
MCP-1/CCL2	monocyte chemoattractant protein 1
M-CSF	macrophage-stimulating factor
MDC	macrophage-derived chemokine
MIG/CXCL9	monokine induced by IFN-γ
MIP-1α/CCL3	macrophage inflammatory protein 1α
PDGF-AA	platelet-derived growth factor AA
RANTES/CCL5	regulated upon activation normal T Cell expressed and presumably secreted
TNF-α	tumoral necrosis factor α
LTA	TNF-β/lymphotoxin alpha
VEGF-A	vascular endothelial growth factor A
NA	Nucleotide analogue.
